# Target Temperature Management Following Pediatric Cardiac Arrest: A Systematic Review and Network Meta-Analysis to Compare the Effectiveness of the Length of Therapeutic Hypothermia

**DOI:** 10.7759/cureus.31636

**Published:** 2022-11-18

**Authors:** Shunsuke Amagasa, Hideto Yasuda, Takatoshi Oishi, Sota Kodama, Masahiro Kashiura, Takashi Moriya

**Affiliations:** 1 Division of Emergency and Transport Services, National Center for Child Health and Development, Tokyo, JPN; 2 Department of Emergency and Critical Care Medicine, Saitama Medical Center, Jichi Medical University, Omiya, JPN; 3 Department of Emergency and Critical Care Medicine, Saitama Medical Center, Jichi Medical University, Saitama, JPN

**Keywords:** post cardiac arrest care, out of hospital cardiac arrest, pediatric intensive care units, post-cardiac arrest syndrome, heart arrest, induced hypothermia

## Abstract

We aimed to compare the efficacy of therapeutic hypothermia for 24, 48, and 72 h, and normothermia following pediatric cardiac arrest. We searched the Cochrane Central Register of Controlled Trials, MEDLINE via Ovid, World Health Organization International Clinical Trials Platform Search Portal, and ClinicalTrials.gov. from their inception to December 2021. We included randomized controlled trials and observational studies evaluating target temperature management (TTM) in children aged < 18 years with the return of spontaneous circulation (ROSC) after cardiac arrest. We compared four intervention groups (normothermia, therapeutic hypothermia for 24 h (TTM 24h), therapeutic hypothermia for 48 h (TTM 48h), and therapeutic hypothermia for 72 h (TTM 72h)) using network meta-analysis. The outcomes were survival and favorable neurological outcome at 6 months or more. Seven studies involving 1008 patients and four studies involving 684 patients were included in the quantitative synthesis of survival and neurological outcome, respectively. TTM for 72 h was associated with a higher survival rate, compared to normothermia (RR 1.75 (95% CI 1.27-2.40)) (very low certainty), TTM 24h (RR 1.53 (95% CI 1.06-2.19)) (low certainty), and TTM 48h (RR 1.54 (95% CI 1.06-2.22)) (very low certainty). TTM for 72 h was also associated with favorable neurological outcomes compared with normothermia (RR 9.36 (95% CI 2.04-42.91)), or TTM 48h (RR 8.15 (95% CI 1.6-40.59)) (all very low certainty). TTM for 24 h was associated with favorable neurological outcome, compared with normothermia (RR 8.02 (95% CI 1.28-50.50)) (very low certainty). In the ranking analysis, the hierarchies for efficacy for survival and favorable neurological outcome were TTM 72h > TTM 48h > TTM 24h > normothermia. Although prolonged therapeutic hypothermia might be effective in pediatric patients with ROSC after cardiac arrest, the evidence to support this result is only weak to very weak. There is no conclusive evidence regarding the effectiveness and length of therapeutic hypothermia and high-quality RCRs comparing long-length therapeutic hypothermia to short-length hypothermia and normothermia are needed.

## Introduction and background

Cardiac arrest often results in death or poor neurological outcome in survivors and is, thus, a significant health burden worldwide. Although cardiac arrest is uncommon in children, their public health burden is high given the potential years of life lost and the length of time that a child may survive with sequelae [1,2]. Hypoxic-ischemic brain injury is the major cause of poor neurological outcomes in survivors of cardiac arrest [3]. As a brain-protective therapy for patients with return of sustained circulation (ROSC), target temperature management (TTM), such as therapeutic normothermia or hypothermia, may be used. TTM is thought to reduce hypoxic-ischemic brain injury by decreasing the cerebral metabolic rate, mitigating reperfusion injury, and inhibiting pathways that lead to neuronal death [4]. The guidelines for post-resuscitation care from the European Resuscitation Council and the American Heart Association recommend TTM for comatose adults after ROSC [5-8]. Therapeutic hypothermia has also been shown to be effective in newborns with birth anoxia [9]. However, it is difficult to directly apply evidence on TTM from newborns with birth anoxia and adults to children after cardiac arrest.

Several observational studies have demonstrated the safety of TTM in children. Randomized controlled trials (RCTs) on the use of TTM in children after cardiac arrest have been published in 2015 and 2017 [10,11]. However, these studies did not demonstrate the efficacy of therapeutic hypothermia in children after a cardiac arrest. A systematic review/meta-analysis published in 2019 did not find any results that supported or rejected the use of hypothermia [12]. Although the duration of therapeutic hypothermia is considered important, previous studies have shown that therapeutic hypothermia is conducted for various durations, including 24 h, 48 h, and 72 h [10,11,13-24]. While current guidelines recommend TTM, there is no mention of the appropriate temperature or length of the treatment [7,8]. Therefore, the comparative effectiveness of different TTM durations in improving survival and neurological outcomes remains unclear.

To compare the efficacy of therapeutic hypothermia of different durations and therapeutic normothermia, we conducted a systematic review and network meta-analysis of observational studies and RCTs to evaluate the relative efficacy of four post-resuscitation target temperatures in comatose patients following cardiac arrest, namely, TTM with normothermia (more than 35.1℃), TTM with induced hypothermia (32-35℃) for 24 h, TTM with induced hypothermia (32-35℃) for 48 h, and TTM with induced hypothermia (32-35℃) for 72 h.

## Review

Materials and methods

Protocol and Registration

This systematic review was designed based on the Preferred Reporting Items for Systematic Review and Meta-Analyses (PRISMA) extension statements for reporting systematic reviews that incorporate network meta-analysis [25]. The review protocol was registered in PROSPERO (CRD42021258215).

Studies, Participants, Interventions/Comparators, and Outcomes

We included RCTs and prospective and retrospective non-randomized studies with a comparator group (non-randomized controlled trials, interrupted time series, controlled before-and-after studies, and cohort studies) in all languages. Cluster randomized trials, crossover trials, case reports or case series, review articles, editorials, and comments were excluded. This meta-analysis included pediatric patients < 18 years of age with ROSC after in-hospital and out-of-hospital cardiac arrest who are in a comatose state. Studies on neonates with perinatal asphyxia were excluded. We included studies that compared at least two of the following four TTM parameters: (1) TTM with normothermia (more than 35.1℃); (2) TTM with induced hypothermia (32-35℃) for 24h (TTM 24h); (3) TTM with induced hypothermia (32-35℃) for 48h (TTM 48h); and (4) TTM with induced hypothermia (32-35℃) for 72h (TTM 72h). We divided the patients into the four intervention groups according to body temperature and TTM length and compared the four groups using a network meta-analysis. Studies with no specifically identified TTM but which reported body temperature were included in the normothermia group thus allowing us to confirm a normal body temperature in patients. Studies that did not report body temperatures and studies with varying TTM times were not included in the quantitative analysis, and qualitative evaluations were conducted.

The primary outcome was the survival rate at discharge or at the longest timepoint described in the studies. The secondary outcome was the rate of a favorable neurological outcome (defined as a Pediatric Cerebral Performance Category 1-3, Cerebral Performance Category 1-2, Glasgow Outcome Scale 4-5, VABS-Ⅱ score ≥70) at the longest timepoint ≥6 months, as described in the studies [26-28].

Data Sources and Search Details

The Cochrane Central Register of Controlled Trials (CENTRAL) and MEDLINE via Ovid were searched for eligible published trials. The World Health Organization International Clinical Trials Platform Search Portal (ICTRP) and ClinicalTrials.gov trial registries were also searched for ongoing trials. If data were missing, we attempted to contact the authors of the study. The search was performed on December 1, 2021. Details of the search strategy and the performed searches are presented in Appendix: Table 10.

Study Selection, Data Collection Process, and Data tems

Citations were documented and duplicates were removed using EndNote software (Thomson Reuters, Toronto, Ontario, Canada). Rayyan software was used for the systematic review. The titles and/or summaries of studies retrieved using the search strategy as well as summaries of studies from additional sources were individually screened by two authors (OT and SK) to determine whether they met the selection criteria described above. The full texts of potentially eligible studies were retrieved and independently assessed for eligibility by two review team members (OT and SK). Any disagreements regarding study eligibility were resolved through discussion with a third reviewer (SA). A standardized pre-pilot form was used to extract data from the included studies and to assess the quality of the studies and synthesis of the evidence. The following information was extracted for analysis: baseline characteristics of the study population and participants, details of the intervention and control conditions, study methodology, outcomes and timepoints of measurement, and information to assess the risk of bias. Two review authors (OT and SK) independently extracted the data, and discrepancies were identified and resolved through discussion with a third author (SA), as necessary.

Risk of Bias Assessment Within Individual Studies

We evaluated the risk of bias in RCTs using the Cochrane Risk of Bias tool 2.0 including bias arising from the randomization process, bias due to deviations from the intended interventions, bias due to missing outcome data, bias in the measurement of the outcomes, and bias in the selection of the reported results [29]. We then classified each study as having low risk, some concerns, or high risk of bias for each of these risk of bias categories. Discrepancies between the two authors were resolved through discussions that included the third author.

We evaluated the risk of bias of non-RCTs using the ROBINS-I tools for non-RCT risk of bias assessment (observational studies) in various domains, namely, bias due to confounding factors, bias in the selection of the study participants, bias in the classification of interventions, bias due to deviations from the intended interventions, bias due to missing data, bias in the measurement of outcomes, and bias in the selection of the reported results [30]. We then classified each study as having low, moderate, serious, or critical risk, or having no information on bias for each item. Discrepancies between the two authors were resolved through a discussion that included the third author.

Statistical Analysis

A network plot was constructed to determine the number of studies and patients included in the meta-analysis. Network meta-analysis (NMA) was performed with the “netmeta 2.0-1” R package (version 4.1.2) using a frequency-based approach with multivariate random effects meta-analysis, and the effect sizes were expressed as RR (95% CI). The certainty of the evidence of the network effect estimate was evaluated based on the following factors using the Grading of Recommendations, Assessment, Development and Evaluation (GRADE) working-group approach of the NMA.

The underlying transitivity assumptions of the NMA were assessed by comparing the distributions of clinical and methodological variables that may serve as effect modifiers across treatment comparisons. The assessment of the risk of bias between studies followed the considerations of pairwise meta-analyses. The conditions associated with "suspicious" and "undetected" bias across studies were determined by the presence of publication bias, as indicated by direct comparisons. The indirectness of each study included in the network was assessed according to its relevance to the research question, consisting of the study population, intervention, outcome, and study setting, and was categorized as low, medium, or high. Study-level judgments could be combined into a contribution matrix. The approach for imprecision involved comparing the range of treatment effects included in the 95% CI with the range of equivalence. The heterogeneity of the treatment effect for clinically important risk ratios (<0.8 or > 1.25) in the CI was assessed. To assess the amount of heterogeneity, we compared the posterior distribution of the estimated heterogeneity variance with its predicted distribution [31]. The CI-based assessment and agreement between the prediction intervals were used to assess the importance of heterogeneity with or without capturing heterogeneity. The heterogeneity of the treatment effect was assessed when the clinically important risk ratio was <0.8 or >1.25 in the prediction interval. Inconsistencies in the network model were estimated from the inconsistency factors and their uncertainty, and consistency was statistically assessed using a design-by-design interaction test [32]. For comparisons informed only by direct evidence, there were no inconsistencies between sources of evidence, and thus “no concern" of inconsistency. When only indirect evidence was included, there was always "some concern.” "Major concern" was considered when the p-value of the interaction test per treatment was <0.01.

As a ranking analysis, the P-score was calculated from the point estimate of the network and the standard error. A treatment's P-score can be interpreted as the average degree of confidence that the treatment is superior to others.

Results

Study Selection

**Figure 1:**
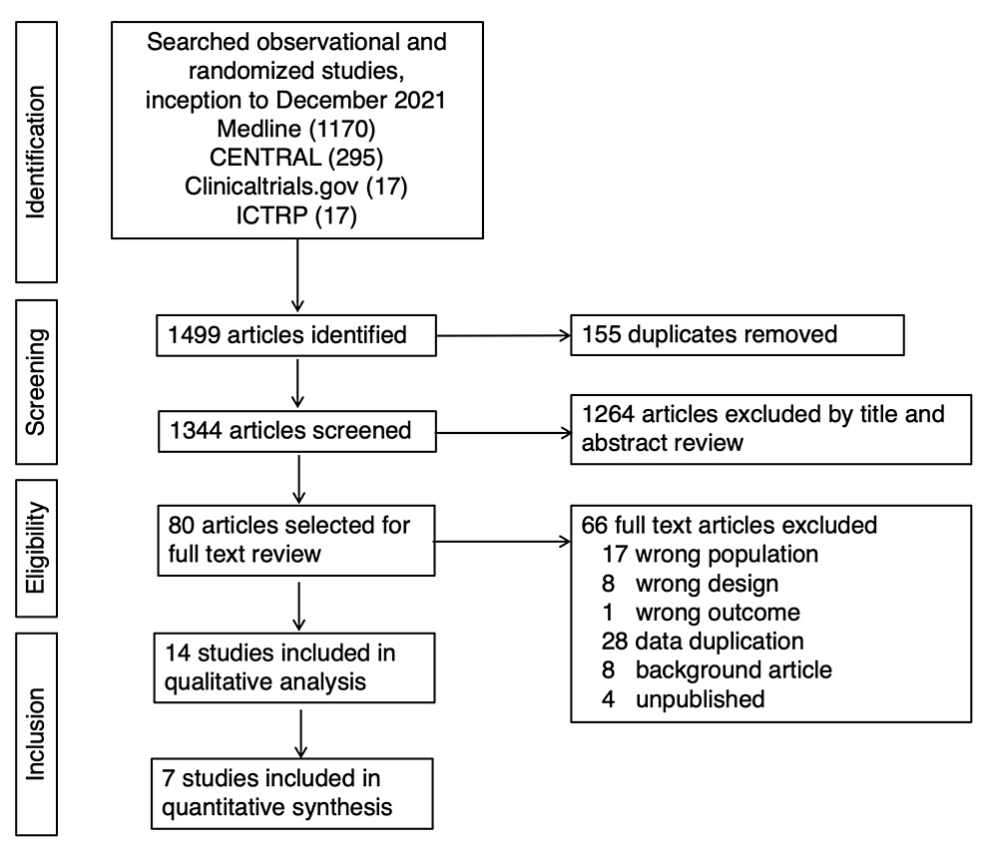
Flowchart of included studies

We identified 1499 articles in the search and screened 1344 articles following the removal of duplicates (Figure 1). Of these, 80 articles were retained for a full-text review. We contacted the investigators of an unpublished study registered on ClinicalTrials.gov. but did not receive an answer. After review, 14 studies [10,11,13-24] were included in the qualitative analysis and 7 studies [10,11,13-17] in the quantitative synthesis (NMA). The reasons for not including 7 studies [18-24] in the quantitative analysis are given in Table 2.

**Table 1:** Study characteristics-1

Study	Survival	Neurological outcome	Narrative summary	Reasons for exclusion from quantitative synthesis
Studies included in quantitative synthesis				
Fink et al., 2010 [13]	Hospital discharge	PCPC at hospital discharge	TTM was not significantly associated with survival and favorable neurological outcome.	NA
Lin et al., 2013 [14]	Hospital discharge	PCPC at hospital discharge	TTM was significantly associated with survival and not significantly associated with favorable neurological outcome.	NA
Scholefield et al., 2015 [15]	Hospital discharge	NA	TTM was not significantly associated with survival. Point estimate was TTM-dominant.	NA
Moler et al., 2015 [10]	12 months	VABS-II score at 12 months	TTM was not significantly associated with survival and favorable neurological outcome. Point estimate was TTM-dominant.	NA
Moler et al., 2017 [11]	12 months	VABS-II score at 12 months	TTM was not significantly associated with survival and favorable neurological outcome.	NA
Lin et al., 2018 [16]	6 months	PCPC at 6 months	TTM was significantly associated with survival and favorable neurological outcome.	NA
Fink et al., 2018 [17]	6 months	PCPC at 6 months	TTM 72h was not significantly associated with survival and favorable neurological outcome. Point estimate for survival was TTM 72h-dominant.	NA
Studies not included in quantitative synthesis but included in qualitative analysis				
Doherty et al., 2009 [19]	6 months	PCPC at 6 months	TTM was not significantly associated with survival and favorable neurological outcome.	Length of therapeutic hypothermia varied.
Buttram et al., 2010 [20]	Hospital discharge	PCPC at hospital discharge	TTM was not significantly associated with survival and favorable neurological outcome. Point estimate was TTM dominant.	Length of therapeutic hypothermia not reported.
van Zellem et al., 2015 [18]	Hospital discharge	NA	TTM was not significantly associated with survival.	Body temperature of no TTM group was not reported.
Chang et al., 2016 [21]	Hospital discharge	CPC at hospital discharge	TTM was not significantly associated with survival and favorable neurological outcome.	Length of therapeutic hypothermia varied.
Cheng et al., 2018 [22]	Hospital discharge	NA	TTM was not significantly associated with survival.	Body temperature of no TTM group was low (mean 34.7℃).
Matsui et al., 2021 [23]	1 month	PCPC at 1 month	TTM was not significantly associated with survival and good neurological outcome.	Length of therapeutic hypothermia varied.
Magee et al., 2021 [24]	NA	Health-related quality of life at 3.8 years (median)	TTM was significantly associated with good neurological outcome.	Length of therapeutic hypothermia varied.

Study Characteristics

The characteristics of the included studies are shown in Tables 1, 2, and 3. Of the 7 studies included in the NMA, 3 were RCTs and 4 were observational studies. The 7 studies included only in the qualitative analyses were observational.

**Table 2:** Study characteristics-2

Study	Years of recruitment	Country of recruitment	Study type	Therapeutic hypothermia	Length	Comparator	Number of patients Treatment/Comparator
Studies Included in quantitative synthesis							
Fink et al., 2010 [13]	2000–2006	US	Observational study	33–35℃	24h	No TTM: 35.4 (33.5–36.3)	40/141
Lin et al., 2013 [14]	2010–2012	Taiwan	Observational study	32–34℃	72h	Normothermia	14/28
Scholefield et al., 2015 [15]	2004–2010	UK	Observational study	32–34℃	24h	TTM<38℃	38/35
Moler et al., 2015 [10]	2009–2012	US	RCT	32–34℃	48h	TTM 36–37.5℃	151/136
Moler et al., 2017 [11]	2009–2015	US	RCT	32–34℃	48h	TTM 36–37.5℃	166/161
Lin et al., 2018 [16]	2010–2017	Taiwan	Observational study	32–34℃	72h	TTM 35.5–37.5℃	25/39
Fink et al., 2018 [17]	2009–2013	US	RCT	32–34℃	24h	TTM 72h	17/17
Studies not being Included in quantitative synthesis but included in qualitative analysis							
Doherty et al., 2009 [19]	2001–2003	Canada and UK	Observational study	<35℃	Variable	No TTM	29/50
Buttram et al., 2010 [20]	2007–2009	US	Observational study	NA	NA	No TTM	33/13
van Zellem et al., 2015 [18]	2002–2011	Netherlands	Observational study	32–34℃	24h	No TTM	63/137
Chang et al., 2016 [21]	2008–2014	Korea	Observational study	32–34℃	Variable	No TTM	81/582
Cheng et al., 2018 [22]	2012–2015	US	Observational study	32–34℃	48h (≧1y), 72h (<1y)	No TTM	26/49
Matsui et al., 2021 [23]	2014–2017	Japan	Observational study	33–36℃	Variable	No TTM	47/120
Magee et al., 2021 [24]	2012–2017	Australia	Observational study	33–34℃	Variable	No TTM	50/78

**Table 3:** Study characteristics-3

Study	Intervention	Number of patients	Age (years) (median, IQR)	Gender, male (%)	Cardiac history	Presumed cardiac cause of arrest	Presumed asphyxia cause of arrest	In-hospital cardiac arrest	Out of hospital cardiac arrest	Initial shockable rhythm	Witnessed arrest	Bystander CPR	Duration of CPR
Fink et al., 2010 [13]	TTM 24h	40	2.4 (0.4-11.8)	24	NA	5	NA	16	24	3	25	NA	15 (10–26)
	No TTM	141	2.9 (1.1-11.1)	80	NA	9	NA	78	63	16	111	NA	8 (3–15)
Lin et al., 2013 [14]	TTM 72h	14	NA	10	2	1	14	6	9	1	NA	NA	mean 20.56±6.35
	No TTM	28	NA	18	0	0	28	8	20	0	NA	NA	mean 24.83±22.2
Scholefield et al., 2015 [15]	TTM 24h	38	1.5 (0-5.8)	17	1	4	NA	0	38	5	23	30	40 (26-56)
	Normothermia	35	1.0 (0-4.0)	8	3	1	NA	0	35	1	22	15	29 (21-46)
Moler et al., 2015 [10]	TTM 48h	151	2.1 (0.6-10.1)	102	14	14	111	0	155	14	58	101	23 (15-35)
	Normothermia	136	1.6 (0.4-7.0)	94	21	18	102	0	140	9	51	85	28 (19-45)
Moler et al., 2017 [11]	TTM 48h	166	1.4 (0.3-5.7)	97	163	89	45	166	0	17	NA	166	23 (7-42)
	Normothermia	161	0.6 (0.2-6.3)	99	146	74	55	163	0	17	NA	163	22 (7-51)
Lin et al., 2018 [16]	TTM 72h	25	NA	21	NA	0	25	0	25	0	12	4	mean 25.56±15.48
	Normothermia	39	NA	28	NA	0	39	0	39	0	25	8	mean 26.00±16.77
Fink et al., 2018 [17]	TTM 72h	17	0.6 (0.3-3.0)	9	NA	1	16	3	14	0	5	13	17 (10-27.5)
	TTM 24h	17	5.7 (0.3-12.7)	5	NA	3	14	3	14	1	7	14	25.5 (17.5-30)

A total of 1008 patients were included in the NMA. The studies were published between 2010 and 2018, with patient recruitment conducted from 2000 to 2017. Three studies exclusively included patients with out-of-hospital cardiac arrest, one exclusively included patients with in-hospital cardiac arrest, and the remaining three studies included mixed populations, regardless of the location where the cardiac arrest occurred. Three different durations of TTM and normothermia were evaluated: 24h (n = 95, 9.4%), 48h (n = 317, 31.4%), 72h (n = 56, 5.6%), and normothermia (n = 540, 53.6%).

Risk of Bias Within Individual Studies

The risk of bias within the included studies is shown in Table 4-7. The risk of bias of 2 RCTs was low in all domains [10,11] and the risk of bias in 1 small RCT was classified as having “some concern” [17]. Among the 5 observational studies, the risk of bias in 3 studies was determined to be “serious”, due to bias in the classification of interventions [13,14,16], with the risk of bias in the other 1 study was determined to be “moderate” [15].

**Table 4:** Risk of bias for survival of randomized controlled trials

Study	Randomization process	Deviations from intended interventions	Missing outcome data	Measurement of the outcome	Selection of the reported result	Overall bias
Moler et al., 2015 [10]	Low	Low	Low	Low	Low	Low
Moler et al., 2017 [11]	Low	Low	Low	Low	Low	Low
Fink et al., 2018 [17]	Low	Some concern	Low	Low	Low	Some concern

**Table 5:** Risk of bias for the neurological outcome of randomized controlled trials

Study	Randomization process	Deviations from intended interventions	Missing outcome data	Measurement of the outcome	Selection of the reported result	Overall bias
Moler et al., 2015 [10]	Low	Low	Low	Low	Low	Low
Moler et al., 2017 [11]	Low	Low	Low	Low	Low	Low
Fink et al., 2018 [17]	Low	Some concern	Low	Low	Low	Some concern

**Table 6:** Risk of bias for survival of observational studies

Study	Bias due to confounding	Bias due to selection participants	Bias in classification interventions	Bias due to deviations from intended interventions	Bias due to missing date	Bias in measurement of outcomes	Bias in selection of the reported result	Overall bias
Fink et al., 2010 [13]	Moderate	Moderate	Serious	Moderate	Low	Low	Low	Serious
Lin et al., 2013 [14]	Serious	Moderate	Serious	Moderate	Low	Low	Low	Serious
Scholefield et al., 2015 [15]	Moderate	Low	Low	Moderate	Low	Low	Low	Moderate
Lin et al., 2018 [16]	Moderate	Moderate	Serious	Moderate	Low	Low	Low	Serious

**Table 7:** Risk of bias for neurological outcome of observational studies

Study	Bias due to confounding	Bias due to selection participants	Bias in classification interventions	Bias due to deviations from intended interventions	Bias due to missing date	Bias in measurement of outcomes	Bias in selection of the reported result	Overall bias
Lin et al., 2018 [16]	Moderate	Moderate	Serious	Moderate	Low	Moderate	Low	Serious

Survival at Discharge or the Longest Timepoint Described in the Studies

**Figure 2:**
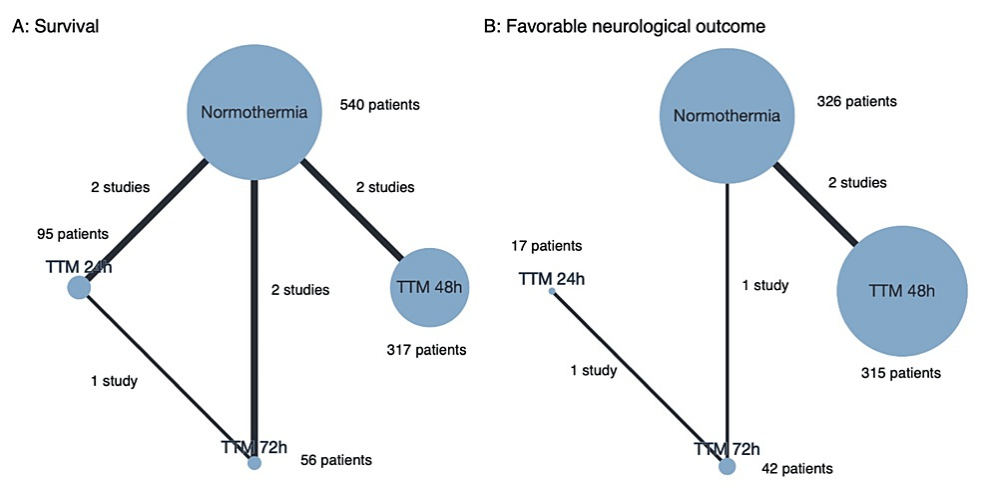
Network plot for A: survival and B: Favorable neurological outcome The node size corresponds to the number of patients who received the intervention. The thickness of the line corresponds to the number of studies that compared the two linked interventions.

**Figure 3:**
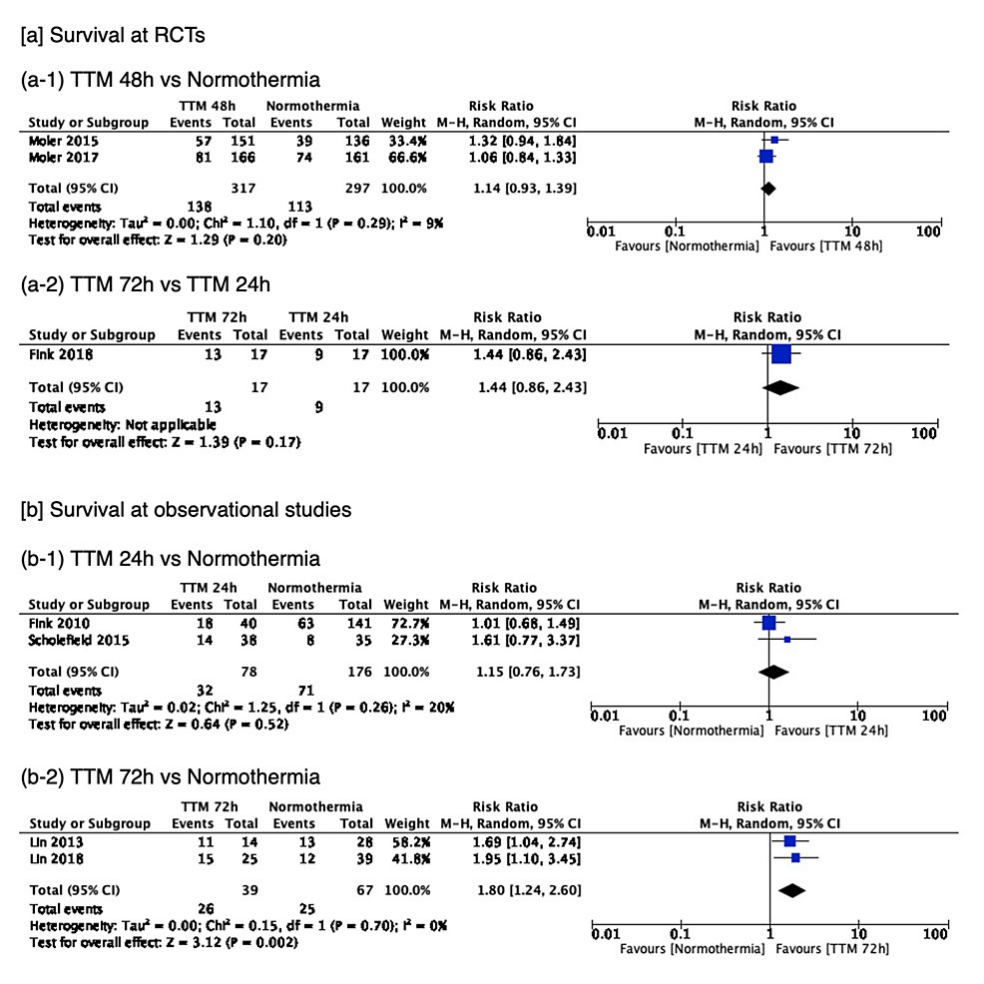
Forest plots for the pairwise comparison of survival TTM 72h was associated with a higher survival rate compared to normothermia. We did not find any association in the other comparisons.

**Figure 4:**
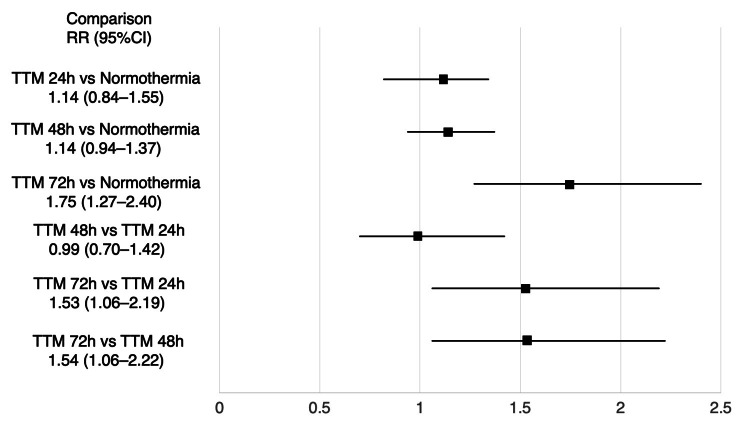
Forest plot of network estimate for survival TTM 72h was associated with survival, compared with no TTM/normothermia, TTM 24h, or TTM 48h. We did not find any association in the other comparisons.

Survival was reported in 7 studies, which included 1008 patients, with the network plot for survival shown in Figure 2. Pairwise comparisons are shown in Figure 3. Pairwise comparison from a small observational study showed that 72h TTM was associated with a significantly higher rate of survival than normothermia, with network estimates shown in Table 8 and Figure 4. TTM 72h was associated with a higher survival rate, compared with normothermia (RR 1.75 (95% CI 1.27-2.40)), TTM 24h (RR 1.53 (95% CI 1.06-2.19)), or TTM 48h (RR 1.54 (95% CI 1.06-2.22)). We did not find any association in the other comparisons. A summary of the confidence in the network estimates is shown in Table 9. The certainty in the network estimates was moderate for TTM 48h vs normothermia, low for TTM 72h vs TTM 24h; very low for TTM 24h vs normothermia, TTM 72h vs normothermia, TTM 48h vs TTM 24h, and TTM 72h vs TTM 48h. The P-scores for survival are shown in Table 10. The hierarchy for efficacy in survival was TTM 72h (P-score 0.99) > TTM 48h (P-score 0.47) > TTM 24h (P-score 0.44) > normothermia (P-score 0.10).

**Table 8:** Network estimates for survival

Comparison	Direct estimate RR (95% Cl)	Indirect estimate RR (95% Cl)	Network estimate RR (95% Cl)	Certainly of evidence for NMA
TTM 24h vs Normothermia	1.12 (0.82–1.34)	1.24 (0.66–2.35)	1.14 (0.84–1.55)	Very low
TTM 48h vs Normothermia	1.14 (0.94–1.37)	NA	1.14 (0.94–1.37)	Moderate
TTM 72h vs Normothermia	1.80 (1.24–2.60)	1.61 (0.86–3.00)	1.75 (1.27–2.40)	Very low
TTM 48h vs TTM 24h	NA	0.99 (0.70–1.42)	0.99 (0.70–1.42)	Very low
TTM 72h vs TTM 24h	1.45 (0.86–2.43)	1.61 (0.97–2.67)	1.53 (1.06–2.19)	Low
TTM 72h vs TTM 48h	NA	1.54 (1.06–2.22)	1.54 (1.06–2.22)	Very low

**Table 9:** Summary of confidence in network estimates for survival

Comparison	Number of studies	Within-study bias	Reporting bias	Indirectness	Imprecision	Heterogeneity	Incoherence	Confidence rating
TTM 24h vs Normothermia	2	Some concerns	Not suggested	No concerns	Some concerns^†^	Some concerns^§^	No concerns	Very low
TTM 48h vs Normothermia	2	No concerns	Not suggested	No concerns	Some concerns^†^	No concerns	No concerns	Moderate
TTM 72h vs Normothermia	2	Major concerns	Not suggested	No concerns	No concerns	No concerns	No concerns	Very low
TTM 48h vs TTM 24h	0	Some concerns	Not suggested	No concerns	Major concerns^‡^	No concerns	No concerns	Very low
TTM 72h vs TTM 24h	1	Some concerns	Not suggested	No concerns	No concerns	Some concerns^§^	No concerns	Low
TTM 72h vs TTM 48h	0	Some concerns	Not suggested	No concerns	No concerns	Some concerns^§^	No concerns	Very low

**Table 10:** P-scores of treatments

Treatment	P-score for survival	P-score for favorable neurological outcome
Normothermia	0.10	0.10
TTM 24h	0.44	0.25
TTM 48h	0.47	0.78
TTM 72h	0.99	0.87

Favorable Neurological Outcome at the Longest Timepoint (≥6 Months)

**Figure 5:**
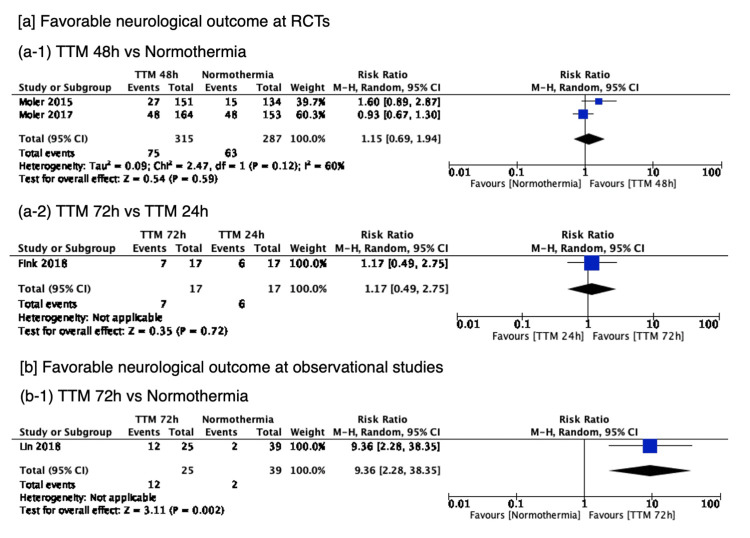
Forest plots for the pairwise comparison of favorable neurological outcomes TTM 72h was associated with a higher rate of favorable neurological outcomes compared to normothermia. We did not find any association in the other comparisons.

**Figure 6:**
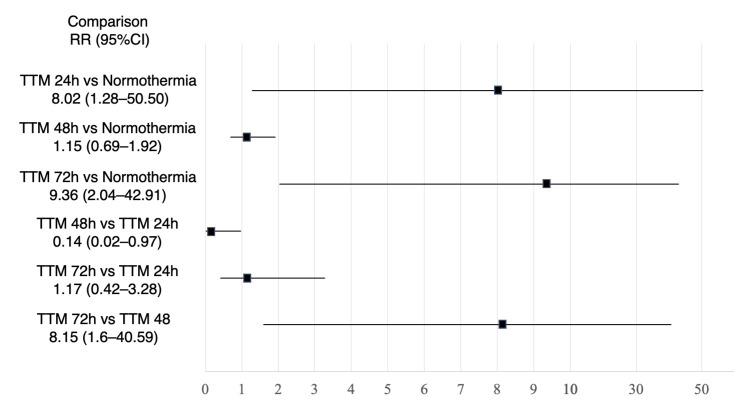
Forest plot of network estimate for favorable neurological outcome TTM 72h was associated with favorable neurological outcomes, compared with no TTM/normothermia, or TTM 48h. TTM 24h was associated with favorable neurological outcomes, compared with no TTM/normothermia. We did not find any association in the other comparisons.

Favorable neurological outcomes at the longest timepoint of ≥6 months were reported in 4 studies including a total of 684 patients, with the network plot for favorable neurological outcomes shown in Figure 2. The pairwise comparisons are shown in Figure 5. Only 1 small observational study showed that 72h TTM was associated with a significantly higher rate of favorable neurological outcome than normothermia. Network estimates are shown in Table 11 and Figure 6. TTM 72h was associated with favorable neurological outcomes, compared with normothermia (RR 9.36 (95% CI 2.04-42.91)), or TTM 48h (RR 8.15 (95% CI 1.6-40.59)). TTM 24h was associated with favorable neurological outcomes, compared with normothermia (RR 8.02 (95% CI 1.28-50.50)). We did not find any association in the other comparisons. A summary of the confidence in the network estimates is shown in Table 12. The certainty in the network estimates was very low for all six comparisons. The P-scores for favorable neurological outcomes are shown in Table 10. The hierarchy for efficacy in favorable neurological outcome was TTM 72h (P-score 0.87) > TTM 48h (P-score 0.78) > TTM 24h (P-score 0.25) > normothermia (P-score 0.10).

**Table 11:** Network estimates of a favorable neurological outcome

Comparison	Direct estimate RR (95% Cl)	Indirect estimate RR (95% Cl)	Network estimate RR (95% Cl)	Certainly of evidence for NMA
TTM 24h vs Normothermia	NA	8.02 (1.28–50.50)	8.02 (1.28–50.50)	Very low
TTM 48h vs Normothermia	1.15 (0.69–1.92)	NA	1.15 (0.69–1.92)	Very low
TTM 72h vs Normothermia	9.36 (2.04–42.91)	NA	9.36 (2.04–42.91)	Very low
TTM 48h vs TTM 24h	NA	0.14 (0.02– 0.97)	0.14 (0.02–0.97)	Very low
TTM 72h vs TTM 24h	1.17 (0.42–3.28)	NA	1.17 (0.42–3.28)	Very low
TTM 72h vs TTM 48h	NA	8.15 (1.6–40.59)	8.15 (1.6–40.59)	Very low

**Table 12:** Summary of confidence in network estimates for favorable neurological outcome

Comparison	Number of studies	Within-study bias	Reporting bias	Indirectness	Imprecision	Heterogeneity	Incoherence	Confidence rating
TTM 24h vs Normothermia	0	Major concerns	Not suggested	No concerns	No concerns	Major concerns^‡^	Major concerns^§^	Very low
TTM 48h vs Normothermia	2	No concerns	Not suggested	No concerns	Major concerns^†^	No concerns	Major concerns^§^	Very low
TTM 72h vs Normothermia	1	Major concerns	Not suggested	No concerns	No concerns	Major concerns^‡^	Major concerns^§^	Very low
TTM 48h vs TTM 24h	0	Some concerns	Not suggested	No concerns	No concerns	Major concerns^‡^	Major concerns^§^	Very low
TTM 72h vs TTM 24h	1	Some concerns	Not suggested	No concerns	Major concerns^†^	No concerns	Major concerns^§^	Very low
TTM 72h vs TTM 48h	0	Some concerns	Not suggested	No concerns	No concerns	Major concerns^‡^	Major concerns^§^	Very low

Discussion

In the present study, 4 forms of TTM after ROSC post-cardiac arrest in children were compared using NMA: normothermia, duration of TTM below 35 °C for 24h, 48h, and 72h. The NMA suggested that TTM for 72h improved survival and neurological outcomes. In addition, the ranking analysis showed that a longer duration of therapeutic hypothermia may be associated with better survival and neurological outcomes. However, this result is largely due to a small number of observational studies showing better survival and neurological outcomes in the TTM 7h group and, therefore, there is only weak evidence to support prolonged therapeutic hypothermia. Overall, there are only a few high-quality studies on TTM after pediatric cardiac arrest and further studies are needed to determine the efficacy and duration of therapeutic hypothermia after pediatric cardiac arrest.

Our NMA showed that TTM 72h may improve survival. Seven studies with survival as an outcome were included in the NMA, and 7 evaluated qualitatively. An RCT (published in 2017) for patients who sustained an in-hospital cardiac arrest (IHCA) found no improvement in survival with 48h of therapeutic hypothermia compared to normothermia [11]. Several observational studies, including those that were not included in the quantitative analysis because of varying therapeutic hypothermia durations, also reported no significant difference in survival between therapeutic hypothermia and normothermia [13,18,19,21-23]. An RCT (published in 2015) of patients who sustained an out-of-hospital cardiac arrest (OHCA) also reported no significant difference in survival between 48h of therapeutic hypothermia and normothermia; however, the point estimate indicated that 48h of therapeutic hypothermia was dominant [10]. In addition, several observational studies also reported point estimate dominance of therapeutic hypothermia [15,20]. Two observational studies suggested that survival was improved after 72h of therapeutic hypothermia compared to normothermia [14,16]. One small RCT reported point estimate dominance of 72h of therapeutic hypothermia compared to 24 h [17]. Our NMA suggested that 72h of therapeutic hypothermia may improve survival, with the ranking analysis also showing a dose-response relationship between the length of therapeutic hypothermia and survival. However, the finding that 72h of hypothermia may be better for survival was largely due to the results of two small observational studies and the treatment effects estimated from the NMA were of low or very low certainty due to possible bias and heterogeneity. Therefore, the effectiveness of 72h of hypothermia is unknown and high-quality RCTs comparing long-length hypothermia to short-length hypothermia and normothermia are needed.

Our NMA showed that TTM 72h may improve neurological outcomes. However, the number of studies assessing neurological outcomes was small and the certainty of the NMA comparison was very low. Quantitative synthesis was only possible for 4 studies that assessed neurological outcomes, while a further 4 studies only be assessed qualitatively. The RCT for OHCA in 2015 and the RCT for IHCA in 2017 both compared therapeutic hypothermia for 48 h to normothermia, finding no significant differences in neurological outcome between hypothermia and normothermia [10,11]. However, consistent with our survival results, the point estimates in the RCT for OHCA suggested that therapeutic hypothermia for 48 h may be beneficial. Two relatively recent observational studies (therapeutic hypothermia 72h vs normothermia or therapeutic hypothermia variable vs. no TTM) have reported improved neurological outcomes with therapeutic hypothermia [16,24], with several studies reporting a dominance of therapeutic hypothermia on point estimates [14,20]. However, other studies did not find any significant differences between therapeutic hypothermia and normothermia [13,17,19,21]. Overall, evidence regarding the effect of therapeutic hypothermia treatment on neurological outcomes in children with ROSC after cardiac arrest is lacking.

Experimental models suggest that a longer length of hypothermia therapy is more useful as a neuroprotective strategy than a shorter length [33-35]. A small RCT comparing 72h versus 24h therapeutic hypothermia after pediatric cardiac arrest reported lower biomarker concentrations of brain injury in the 72h therapeutic hypothermia group [17]. In neonates, 72h therapeutic hypothermia has been shown to be effective for hypoxic encephalopathy [36]. Overall, there were no high-quality studies on the length of therapeutic hypothermia following cardiac arrest in children future studies on the benefit of prolonged therapeutic hypothermia are needed.

A problem in studies on cardiac arrest is the heterogeneity of patients included [12,37,38]. There are differences between OHCA and IHCA not only in terms of the location of the cardiac arrest but also in the likelihood of etiology and the presence of underlying diseases, amongst other factors [39,40]. OHCA is often respiratory in origin, whereas IHCA is often cardiac in origin and occurs in children with underlying diseases. Although there is no major difference in the treatment after resuscitation, the time to treatment also differs between OHCA and IHCA patients. The usefulness of therapeutic hypothermia has been reported to be greater for certain patient populations [41]. In our study, OHCA and IHCA were also present and their etiology varied; however, sub-analysis was not possible due to the small number of studies and patients and the inability to collect data by etiology and location of cardiac arrest. Future studies on the assessment and quantitative synthesis of the efficacy of therapeutic hypothermia in terms of the location and etiology of the cardiac arrest, together with the duration and temperature of the TTM, are needed.

Strengths and Limitations

To the best of our knowledge, there were no studies to compare the effectiveness of the duration of therapeutic hypothermia for children with ROSC after cardiac arrest using quantitative integration with NMA. Although previous meta-analyses have not reported conclusive findings either supporting or rejecting the use of therapeutic hypothermia in pediatric cardiac arrest, our study observed that longer therapeutic hypothermia, especially TTM for 72 h, may improve outcomes. A broad search was conducted for observational studies and RCTs without language restrictions, and qualitative evaluation and quantitative synthesis, using NMA, were conducted for the included studies.

However, the limitations of our study need to be acknowledged. First, quantitative synthesis included RCTs and observational studies without fully adjusting for confounders. Most of the comparisons on the certainty of evidence for NMA were “low” or “very low” due to the inclusion of observational studies and their inherent confounders and biases. Despite this limitation, we believe that our study is important as a pilot study to evaluate the effects of the length of therapeutic hypothermia. Second, we did not compare the temperatures of the therapeutic hypothermia in this study because there was not much difference in the therapeutic hypothermia because there was not much difference in the therapeutic hypothermia temperatures used in the different studies. This could be an effect modifier or an interaction. Third, certain comparisons had only direct or indirect data. Future studies comparing the duration of hypothermia and NMAs that include such studies are needed.

## Conclusions

Although prolonged therapeutic hypothermia might be effective in pediatric patients with ROSC after cardiac arrest, the evidence to support this result is only weak to very weak. There is no conclusive evidence regarding the effectiveness and length of therapeutic hypothermia. High-quality RCTs comparing long-length therapeutic hypothermia to short-length hypothermia and normothermia are needed.

## Appendices

**Table 13:** Search strategy

MEDLINE via OVID	1. exp Heart Arrest/ 2. exp Ventricular Fibrillation/ 3. exp Tachycardia, Ventricular/ 4. exp Cardiopulmonary Resuscitation/ 5. exp Heart Massage/ 6. exp Return of Spontaneous Circulation/ 7. exp Post Cardiac Arrest Syndrome/ 8. neuroprotection.mp. 9. (asystole or dysrhythmia or ((heart or cardiac or ventricular or circulatory) adj3 arrest) or (ventricular adj5 (fibrillation or tachycardia or arrhythmia)) or "pulseless electrical activity" or "electromechanical dissociation" or "cardiopulmonary resuscitation" or (PEA or EMD) or "non‐perfusing rhythm").mp. 10. (acls or als or (advanced adj3 support$)).tw. 11. 1 or 2 or 3 or 4 or 5 or 6 or 7 or 8 or 9 or 10 12. exp Pediatrics/ or exp Child/ or exp Infant/ or exp Adolescent/ or (infan$ or newborn$ or new-born$ or perinat$ or neonat$ or baby or baby$ or babies or toddler$ or minors or minors$ or boy or boys or boyfriend or boyhood or girl$ or kid or kids or child or child$ or school or school$ or adolescen$ or juvenil$ or youth$ or teen$ or under$age$ or pubescen$ or pediatric$ or paediatric$ or peadiatric$ or prematur$ or preterm$).mp. 13. 11 and 12 14. exp Cryotherapy/ 15. exp Body Temperature/ 16. exp Hypothermia/ 17. (hypotherm$ or normotherm$ or cool$ or cold$ or temperature$ or cryother$ or cryogen$ or cryotreat$ or refrigeration or "artificial hibernation").ti,ab. 18. 14 or 15 or 16 or 17 19. randomi?ed.ti,ab. 20. randomized controlled trial.pt. 21. controlled clinical trial.pt. 22. placebo.ab. 23. clinical trials as topic.sh. 24. randomly.ab. 25. trial.ti. 26. Comparative Study/ 27. 19 or 20 or 21 or 22 or 23 or 24 or 25 or 26 28. (animals not (humans and animals)).sh. 29. 27 not 28 30. exp cohort studies/ 31. cohort$.tw. 32. controlled clinical trial.pt. 33. epidemiologic methods/ 34. limit 33 to yr=1966-1989 35. exp case-control studies/ 36. (case$ and control$).tw. 37. observatio$.tw. 38. 30 or 31 or 32 or 34 or 35 or 36 or 37 39. 29 or 38 40. 13 and 18 and 39
CENTRAL	1. MeSH descriptor: [Pediatrics] explode all trees 2. MeSH descriptor: [Child] explode all trees 3. MeSH descriptor: [Infant] explode all trees 4. MeSH descriptor: [Adolescent] explode all trees 5. (infan* OR newborn* OR new-born* OR perinat* OR neonat* OR baby* OR babies OR toddler* OR minors* OR boy OR boys OR boyfriend OR boyhood OR girl* OR kid OR kids OR child* OR school* OR adolescen* OR juvenil* OR youth* OR teen* OR under*age* OR pubescen* OR pediatric* OR paediatric* OR peadiatric* OR prematur* OR preterm*):ti,ab,kw 6. 1 OR 2 OR 3 OR 4 OR 5 7. MeSH descriptor: [Heart Arrest] explode all trees 8. MeSH descriptor: [Ventricular Fibrillation] explode all trees 9. MeSH descriptor: [Tachycardia, Ventricular] explode all trees 10. MeSH descriptor: [Cardiopulmonary Resuscitation] explode all trees 11. MeSH descriptor: [Heart Massage] explode all trees 12. MeSH descriptor: [Return of Spontaneous Circulation] explode all trees 13. MeSH descriptor: [Post-Cardiac Arrest Syndrome] explode all trees 14. (neuroprotection):ti,ab,kw 15. (asystole OR dysrhythmia OR ((heart OR cardiac OR ventricular OR circulatory) NEAR/3 arrest) OR (ventricular NEAR/5 (fibrillation OR tachycardia OR arrhythmia)) OR "pulseless electrical activity" OR "electromechanical dissociation" OR "cardiopulmonary resuscitation" OR (PEA OR EMD) OR "non‐perfusing rhythm"):ti,ab,kw 16. (acls OR als OR (advanced NEAR/3 support*)):ti,ab,kw 17. 7 OR 8 OR 9 OR 10 OR 11 OR 12 OR 13 OR 14 OR 15 OR 16 18. 6 AND 17 19. MeSH descriptor: [Cryotherapy] explode all trees 20. MeSH descriptor: [Body Temperature] explode all trees 21. MeSH descriptor: [Hypothermia] explode all trees 22. ((hypotherm* OR normotherm* OR cool* OR cold* OR temperature* OR cryother* OR cryogen* OR cryotreat* OR refrigeration OR "artificial hibernation")):ti,ab,kw 23. 19 OR 20 OR 21 OR 22 24. 18 AND 23
Clinicaltrials.gov	INFLECT EXACT "Alll Studies" [STUDY-TYPES] AND (asystole OR ((heart OR cardiac OR circulatory) AND arrest) OR (ventricular AND (fibrillation OR tachycardia)) OR "pulseless electrical activity" OR "electromechanical dissociation" OR "cardiopulmonary resuscitation" OR pea OR emd)[DISEASE] AND (hypothermia OR normothermia OR cool OR cooling OR cold OR temperature OR cryotherapy OR cryogenic OR refrigeration OR “artificial hibernation”)[TREATMENT] AND Child (birth-17)[AGE GROUP]
ICTRP	Condition: (asystole OR ((heart OR cardiac OR circulatory) AND arrest) OR (ventricular AND (fibrillation OR tachycardia)) OR "pulseless electrical activity" OR "electromechanical dissociation" OR "cardiopulmonary resuscitation" OR pea OR emd OR "non-perfusing rhythm") AND Intervention: (hypotherm* OR normotherm* OR cool* OR cold* OR temperature* OR cryother* OR cryogen* OR cryotreat* OR refrigeration OR "artificial hibernation”)
